# Do Thyroid Disrupting Chemicals Influence Foetal Development during Pregnancy?

**DOI:** 10.4061/2011/342189

**Published:** 2011-09-11

**Authors:** Marie-Louise Hartoft-Nielsen, Malene Boas, Sofie Bliddal, Åase Krogh Rasmussen, Katharina Main, Ulla Feldt-Rasmussen

**Affiliations:** ^1^Department of Medical Endocrinology PE-2131, Rigshospitalet, University Hospital of Copenhagen, 2100 Copenhagen, Denmark; ^2^Department of Growth and Reproduction, Rigshospitalet, University Hospital of Copenhagen, 2100 Copenhagen, Denmark

## Abstract

Maternal euthyroidism during pregnancy is crucial for normal development and, in particular, neurodevelopment of the foetus. Up to 3.5 percent of pregnant women suffer from hypothyroidism. Industrial use of various chemicals—endocrine disrupting chemicals (EDCs)—has been shown to cause almost constant exposure of humans with possible harmful influence on health and hormone regulation. EDCs may affect thyroid hormone homeostasis by different mechanisms, and though the effect of each chemical seems scarce, the added effects may cause inappropriate consequences on, for example, foetal neurodevelopment. This paper focuses on thyroid hormone influence on foetal development in relation to the chemicals suspected of thyroid disrupting properties with possible interactions with maternal thyroid homeostasis. Knowledge of the effects is expected to impact the general debate on the use of these chemicals. However, more studies are needed to elucidate the issue, since human studies are scarce.

## 1. Introduction

Maintaining maternal euthyroidism during pregnancy is important for growth and development, in particular neurodevelopment of the foetus. Even subtle changes in thyroid function of the pregnant woman can cause detrimental effects for the foetus [1–5]. In the first trimester, the foetus relies solely on the thyroid hormones thyroxine (T4) and tri-iodothyronine (T3) and iodine from the mother. Later in pregnancy and during lactation, maternal thyroid hormones still contribute significantly to foetal thyroid homeostasis [6–8]. Worldwide, both overt and subclinical hypothyroidism are frequent among fertile women [9–14]. Prior maternal thyroid diseases as well as iodine and selenium deficiencies are known risk factors for hypothyroidism. 

Abundant industrial and household use of various chemicals—called endocrine disrupting chemicals (EDCs)—expose humans with potential harmful influences on health and hormone regulation. As recently reviewed, several of these EDCs have been found to have thyroid disrupting properties as well [15–17]. Probably each chemical has limited thyroid disruptive effects at environmental exposure doses. However, the combined influence of several chemicals through different pathways of thyroid hormone synthesis and action may have significant impact on both maternal and foetal thyroid function [18, 19] and, thus, a potential to compromise foetal development and maturation. 

This paper will focus on the influence of thyroid hormones on foetal development in relation to the chemicals suspected to have thyroid disrupting properties. Knowledge on these effects is expected to impact international debate on the general use of these chemicals.

## 2. Maternal and Foetal Thyroid Status during Pregnancy

**Figure 1:**
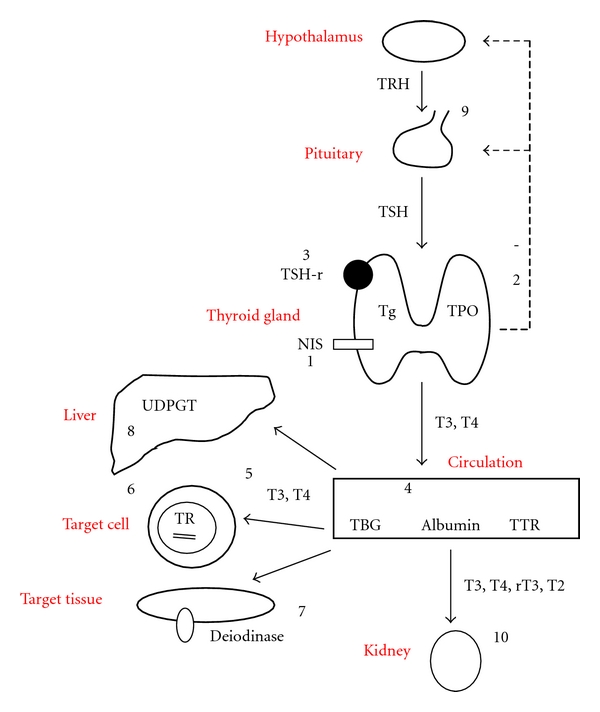
The complex mechanisms of regulation of thyroid hormone homeostasis and the possible mechanism of action of the thyroid disrupting chemicals. The thyroid and the thyroid hormones, tri-iodothyronine (T3) and thyroxine (T4), participate with the hypothalamus, secreting thyrotropin releasing hormone (TRH), and pituitary, secreting thyrotropin (TSH) in a classical feedback controlled loop. Iodide is transported into the cell by the sodium-iodine symporter (NIS) and oxidized by thyroid peroxidase (TPO). TPO also catalyzes the iodination of thyrosine residues on thyroglobulin (Tg). All processes in the cell are stimulated by binding of TSH to the TSH receptor (TSH-R). In the circulation, thyroid hormones are bound to thyroxine-binding globulin (TBG), albumin and prealbumin, and in some cases transthyretin (TTR). T4 is deiodinated by deiodinases in the liver and target tissues. In the target cells, T3 binds to nuclear thyroid hormone receptor (TR), and with the retinoid X receptor, it binds at specific sequences at the DNA string, forming the thyroid hormone response elements (TRE). In the liver, thyroid hormones are metabolized by UDP-glucuronyl transferase (UDPGT), and finally, the metabolites are excreted in the urine. (1) Inhibition of iodine uptake in the cells by inhibition of NIS: perchlorate, thiocyanate, nitrate, and phthalates. (2) TPO inhibition: NP and isoflavones. (3) Inhibition of TSH-R: DDT and PCB. (4) Binding to transport proteins: PCB, phthalates, phenol, flame retardants, and HCB. (5) Cellular uptake of thyroid hormones: phthalates and chlordanes. (6) Binding to thyroid hormone receptor and affecting gene expression: PCB, phenols, flame retardants, BPA and HCB. (7) Inhibition of deiodinases: Styrenes and UV-filters, (8) Activation of hepatic UDPGT: dioxins and pesticides, (9) Inhibition of the hypothalamo-pituitary-thyroid axis: lead. (10) Excretion/clearance of thyroid hormones: PCB, dioxin, phenols, flame retardants, HCB, and BPA.

The main task of the thyroid gland is to generate the necessary quantity of thyroid hormone to meet the demands of the organism. The mechanisms involved in thyroid homeostasis are shown in Figure 1. Each step of thyroid hormone metabolism is crucial for normal function. Maternal thyroid status is subject to substantial pregnancy-related physiological changes. Importantly, maternal thyroid hormone is metabolized by or crosses the placenta to reach the foetus [20]. In the placenta, the inner ring placental deiodinase inactivates most of the maternal T4 to reverse T3 (rT3), securing a minimal but highly significant supply of thyroid hormones to the foetus [20, 21], which further demands an increased thyroid hormone production by the mother. 

The foetal thyroid function is established in the 11th week after conception [6]. However, the production and secretion of foetal thyroid hormones do not reach notable levels until midgestation [6]. Even at term, up to 30% of the foetal thyroid hormones are of maternal origin [22], and during the remaining part of pregnancy and lactation, the foetus and neonate are strongly dependent on the maternal thyroid gland.

## 3. Influence of Maternal Thyroid Disease on Foetal Development

The estimated prevalence of overt and subclinical hypothyroidism in pregnancy is 0.5% and 3%, respectively. Thyroid autoantibodies are found in 5%–15% of women of childbearing age [9–14]. The estimated high prevalence of thyroid disease in pregnant women has spurred a debate of whether screening of all pregnant women, instead of only targeted case-finding, should be advised. In recent studies, 50% to 80% of the pregnant women with possible hypothyroidism would be missed if only high-risk cases were examined [23, 24], but screening of all pregnant women is not yet agreed upon in international scientific associations [25]. 

At least 50% of the offspring of women with free T4 (fT4) levels below the normal 10th percentile had delayed neurobehavioral development [2, 3, 26]. Even mild-to-moderate iodine deficiency during first trimester caused an intelligence quotient (IQ) 10–15 points below the normal mean and 11 of 16 children born to mothers with low iodine intake presented attention deficit hyperactivity disorders [27]. Iodine deficiency is the most frequent cause of maternal hypothyroxinaemia and a potentially preventable cause of mental retardation in children.

## 4. Endocrine Disrupting Chemicals and the Thyroid Gland

In recent years, numerous chemicals have been shown to interfere at different levels of thyroid hormone regulation and function (Figure 1). Most chemicals have not yet been sufficiently evaluated in humans. Yet, a number of detrimental effects on human thyroid function are suspected from a variety of chemicals, and a review of available evidence on this issue will be focused upon in the following. 

### 4.1. Perchlorate

Perchlorate is a persistent ubiquitous chemical used worldwide in nitrate fertilizers, fireworks, road flare, matches, airbag inflation systems, and as oxidizers in solid propellants for rockets and missiles. Perchlorate appears in drinking water, milk, wine, beer, and lettuce, but also a natural perchlorate background of atmospheric origin exists [28]. Perchlorate has previously been used in the treatment of hyperthyroidism [29] due to its potent competitive inhibition of thyroid iodine uptake through the sodium-iodine symporter (NIS) [30]. However, the thyroid disrupting effect of perchlorate is dose dependent. Thus, occupational or environmental exposures of perchlorate have been associated with a reduction in thyroid iodine uptake [31–33] but without direct effects on thyroid function or volume except in a study of women with urinary iodine excretion below 100 *μ*g/L in whom TSH was increased and TT4 was found reduced [34], and these findings are further supported by findings of an interaction of perchlorate and thiocyanate on thyroid status in smoking women with low iodine intake [35] (Table 1). A study of euthyroid and hypothyroid pregnant women from Cardiff in Wales and Turin in Italy found perchlorate in all urine samples and low iodine excretion from all the pregnant women, but no correlation was found between perchlorate levels and thyroid function parameters [36]. Likewise, in pregnant women and their neonates, perchlorate in drinking water did not influence thyroid hormone levels [37, 38], and no correlations were found between urinary perchlorate concentrations and fT4 or thyroid stimulating hormone (TSH), respectively, during first trimester in mildly hypothyroid women. Iodine is secreted into breast milk through NIS, and one study found that the highest concentrations of perchlorate in breast milk were associated with lower iodine concentrations [39], while others found no obvious correlations [40].

### 4.2. Thiocyanate and Nitrate

Thiocyanate and nitrate are less potent inhibitors of NIS than perchlorate [30] but, nitrate may decrease iodine absorption from the intestine [47].

Thiocyanate is present in a number of vegetables such as cabbage, broccoli, Brussels sprouts, rapeseed and mustard seed, cassava, radishes, spinach and tomatoes but also in milk. In many tropical countries, cassava as staple food is a major ingredient in the daily food supply. In iodine-deficient regions, food with high concentrations of thiocyanate contributes significantly to goitre development [48, 49]. However, in industrialized societies, the main source of thiocyanate is cigarette smoke [48]. Although this has well-known detrimental effects on the thyroid function of neonates and breastfed babies, it is beyond the scope of this paper.

Nitrate is found in several food items either occurring naturally, as in green leafy vegetables, or added as a preservative in cubed meats and other food and is also generated from the decomposition of organic materials. Inorganic nitrates are used as fertilizers, which may contaminate drinking water supplies, groundwater, and soil. Finally, the intestinal flora causes an endogenous formation of nitrate. Population studies on nitrate exposure through drinking water have found increased thyroid volume and slightly reduced thyroid function [50], but the isolated effect of nitrate has been difficult to assess due to concomitant iodine deficiency [51, 52]. But low levels of nitrate intake did not influence thyroid volume in adults despite of previous iodine deficiency [53].

### 4.3. Polychlorinated Biphenyls (PCBs)

PCBs are still in use though several of them have been banned for decades in many countries. PCBs and their hydroxylated metabolites are biologically active, highly persistent compounds accumulating in lipid tissues, and structurally very close to T4 [54]. Many studies have been performed on the thyroid disturbing effects of PCBs, but results are conflicting (Table 2). PCBs may interfere with thyroid hormone homeostasis in several ways (Figure 1): by binding to transthyretin (TTR) [55], by affecting the expression of thyroid hormone-responsive genes, and by antagonizing the complexes from the thyroid hormone responsive elements (TRE) [56, 57]. Perinatal exposure may be most important in humans. Negative correlations have been demonstrated between PCBs in maternal blood during pregnancy and maternal thyroid hormones, and positive correlations have been described between PCBs and TSH [58]. As thyroid hormones in humans are mainly bound to thyroid hormone-binding globulin (TBG), the reduction in total T4 (TT4) and total T3 (TT3) could be explained by a reduced TBG level, whereas this would not necessarily affect free hormone levels [59]. In cord blood, a positive correlation of PCB and TSH of the child and a negative correlation with maternal TT3 and TT4 were found [60]. PCBs in cord blood have generally not demonstrated associations to T3 and T4 levels of the child [58, 61–65], except in a recent study finding higher TSH and lower T4 in infants of mothers with high levels of PCB in breast milk [66, 67]. Yet, not all studies found associations between infant thyroid hormone levels and PCB exposure [63–65, 68], and in a study of a prenatal boys exposed to high PCB levels, the thyroid function was comparable to that of the control group [69]. 

In several studies of humans of all ages from high PCB-exposed areas, blood PCB concentrations correlated negatively to circulating thyroid hormone levels [76, 79, 80, 83] and positively to TSH [74], while others could not find such associations [78, 81]. Increased thyroid volume has also been found more often in a PCB-polluted area with the largest volumes among subjects with the highest levels of PCB [82].

### 4.4. Dioxin

Dioxins are highly toxic, lipophilic, widely used, and persistent environmental pollutants from industrial burning processes or production of herbicides, detectable in samples from humans and wildlife populations though banned for years in many countries. The most toxic prototype is 2,3,7,8-tetrachlorodibenzo-p-dioxin (TCDD), and the toxic equivalent of all other dioxins is measured against this. In particular, the metabolites show a high degree of structural similarity to T4 and are the most biologically active. Dioxins have been found to decrease the level of circulating thyroid hormones in rats [85–87], and mixtures of dioxin-like compounds were even found to reduce levels of T4 in an additive manner [88]. Given to pregnant rats, a single dose of TCDD was transferred to the pups via placenta and during lactation [89] and resulted in a dose-dependent decrease of T4 and fT4 with a concomitant increase in TSH [86, 87]. High exposure with TCDD of US war veterans of the Vietnam war resulted in significantly increased TSH [90]. In children, no associations between placental dioxins and thyroid hormones were found at the age of 2 years, but after 5 years, T3 was significantly higher in the highly exposed individuals in utero [91]. But as recently reviewed, so far, no clear and significant correlation between background exposure to dioxins and thyroid function during development has been found [92].

### 4.5. Phthalates

Phthalates are widely used chemicals mainly to improve the flexibility of materials such as plastic and have been widely used in medical products, food handling and storage products, electrical devices, toys, and in non-polyvinylchloride applications such as paints, lacquers, and cosmetics. Phthalates can leach, migrate, or evaporate into indoor air and atmosphere, foods, and liquids and have become ubiquitous. Consequently, humans are constantly exposed by oral, inhalation, and dermal routes [93]. Unfortunately, certain vulnerable groups may be massively exposed to phthalates, such as hospitalized neonates in whom urinary excretion of phthalates was shown to correlate with exposure to medical devices [94]. However, a followup of adolescents exposed to high concentrations of phthalates in the neonatal period showed normal thyroid hormones [95]. On the other hand, men recruited from a fertility clinic [96] and pregnant women [97] demonstrated a negative association between phthalates and fT4 and T3, respectively. 

We studied 845 children aged 4–9 years with determination of urinary concentrations of 12 phthalate metabolites and serum levels of TSH, thyroid hormones, and insulin-like growth factor-I (IGF-I) [98]. Our study showed a negative association between urinary phthalate concentrations and thyroid hormones, IGF-I and growth of the children, respectively. Although our study was not designed to reveal the mechanism of action, the overall coherent negative associations may suggest causative negative roles of phthalate exposures for child health.

### 4.6. Triclosan and Bisphenol A

The exact thyroid disturbing mechanisms of these chemicals are not known, but triclosan, and bisphenol A (BPA) share structural similarities with thyroid hormones and may bind to and interact with the thyroid hormone receptor (TR). Phenols bind competitively to TTR, [99, 100] and act as a T3 antagonist [101, 102].

BPA is used to manufacture polycarbonate and several hard plastic products such as compact discs, food can linings, adhesives, powder paints, dental sealants, and clear plastic bottles which means that humans are ubiquitously exposed to BPA [103, 104]. BPA is rapidly glucuronidated in humans and rodents. 

Phenols were found to bind competitively to TTR, possibly with a very strong binding affinity [99, 100], but a recent study found that the concentrations of BPA usually found in humans is probably not high enough to interfere with T4 transport [105]. Finally, T3-mediated gene activation through TR*α*1 and TR*β* was dose-dependently suppressed by, BPA and the expression of T3- suppressed genes was up-regulated by BPA [101, 102]. In pregnant rats, BPA was associated with a significant increase of TT4 in the pups 15 days postpartum [106]. 

Triclosan in an antibacterial and antifungal agent used in products for personal hygiene and household cleaning agents but also in plastics and fabrics. Though found in human urine [107] and breast milk [108], so far, no epidemiological studies have been published on the influence of triclosan on thyroid hormone homeostasis. A small intervention study [109] could not demonstrate changes in CYP3A4-activity or peripheral thyroid hormone levels after triclosan exposure through toothpaste. However, in vitro studies suggest that higher exposure levels may activate human pregnane x receptor, which upregulates the activity of CYP3A4 [110]. In rats, gestational exposure to triclosan lowered T4 in the pregnant animal and transitorily in the pups at postnatal day 4 [111, 112].

### 4.7. Isoflavones

Isoflavones, naturally occurring phytoestrogens, are mainly found in soy and grain products [113]. Isoflavones inhibit thyroid peroxidase (TPO) function and thereby thyroid hormone production [114]. Iodine insufficient children fed on soy products risk development of goitre and hypothyroidism [115]. As reviewed by Messina and Redmond several studies have been performed in humans to explore the thyroid disrupting effect of isoflavones, but only one study from Japan of healthy volunteers fed for 1–3 months with soy beans reported increased TSH though within the normal reference interval and increased thyroid volume. But other studies could not reveal such relationships [116].

### 4.8. Brominated Flame Retardants

Flame retardants constitute a group of chemicals such as tetrabromobisphenol A (TBBPA), a halogenated derivative of BPA and polybrominated biphenyls. These chemicals are found in different products such as plastic paints and synthetic textiles and are often used in electrical devices such as televisions, computers, copying machines, video displays, and laser printers. These chemicals are structurally more similar to T4 than PCBs and bind competitively to TTR [99]. In general, flame retardants are found to reduce thyroid hormone levels. A recently published study of pregnant women showed a negative association between serum levels of brominated flame retardants and TSH [117]. A newer study of recreational fish consumers reported a negative association between concentrations of some congeners in serum and serum levels of T3 and TSH and a positive relationship with T4 [118]. This was confirmed by others [78] but not all [119], and in a smaller study of 12 mother-infant pairs, maternal brominated flame retardants levels were not significantly correlated to thyroid hormone levels in cord blood [120].

### 4.9. Pesticides

Pesticides constitute a large and very inhomogeneous group of chemicals, which differ significantly in their chemical and physical properties and, thus, their ability to be either detoxified in vivo or to bioaccumulate in lipid-rich tissue. It is beyond the scope of the paper to give a comprehensive overview about potential thyroid disrupting effects. Many of the organochlorine pesticides are persistent with long environmental half-lives, and therefore, humans are continuously exposed though many pesticides have been banned for years in many countries while still in use in others. Dichlorodiphenyltrichloroethane (DDT), hexachlorobenzene (HCB), and nonylphenol (NP) are among the most examined. Metabolites of HCB are used as a biocide and wood preservative in the timber industry and as antifungal agent in the leather industry. NP is an industrial additive used in detergents, plastics, and pesticides. In humans, an enlarged thyroid was found after accidental exposure to HCB [121], and studies have found negative associations between HCB and T4 [77, 81] or T3 [58] but not TSH or free thyroid hormone levels [77]. In newborns, pentachlorphenol (PCP) in cord blood but not HCB [58] was negatively correlated to T3, fT4 and TBG [122], and thus may potentially impair neurodevelopment. Also, other pesticides seem to posses thyroid disrupting properties [123–127].

### 4.10. Others

Ultraviolet (UV) filters also called sunscreens, that is, benzophenone, 4-methylbenzylidene camphor and 3-benzylidene camphor, comprise a group of chemicals used to absorb and dissipate UV irradiation in cosmetic products, not only sun lotions, to enhance product longevity and quality. So far, only animal and in vitro studies have indicated that UV filters may disrupt thyroid hormone homeostasis. 

Parabens are commonly used as preservatives in food, cosmetics and pharmaceutical products. In vitro methyl-paraben dose-dependently inhibited iodine organification and thus seemed to have a weak intrinsic antithyroid effect [128], but human studies are lacking. 

The industrial use of perfluorinated chemicals (PFC) is increasing in products such as stain- and oil-resistant coatings for example, food packaging for fast food, as well as in floor polishes and insecticide formulations. PFCs are extremely persistent in the environment. Women with high levels of PFCs were treated more often for thyroid disease than controls [129], and in employees from a PFC factory, PFCs displayed a negative association to fT4 [130].

Styrene is an industrial chemical widely used in the production of plastics, resins, and polyesters. Humans are exposed by low-level contamination in food items, but the exposure is most abundant through inhalation of tobacco smoke, automobile exhaust, and vapors from building materials [131]. Occupational styrene exposure resulted in thyroid disrupting effects: there was a positive correlation between exposure time and thyroid volume and a positive correlation between urinary concentrations of styrene metabolites and f T4 or fT4/fT3 ratios without a correlation to TSH. This indicated an inhibition of the conversion of T4 to T3 [132].

Exposure to lead is typically from cigarette smoke or gasoline, but also workers in the mining, smelting, refining, battery manufacturing, soldering, electrical wiring, and ceramic glazing industries are at risk of occupational exposure. Lead may cause a toxic effect on the central part of the hypothalamic-pituitary-thyroid axis [133, 134], but the mechanism is not yet known and effects on the selenium metabolism is also possible. In lead-exposed children, an impaired release of TSH has been reported [135], but another study found unchanged T4 levels after lead exposure [136]. 

Studies in occupational lead exposed workers indicates induction of secondary hypothyroidism; one study found low T4 and fT4 and inappropriately normal TSH [137] and in auto repair workers, a negative correlation between blood lead levels and fT4 was found, but TSH, T3, and thyroid volume were comparable to unexposed controls [133]. In another group of petrol pump workers or mechanics, TSH was increased compared to the unexposed controls, and T3 declined by longer exposure, but T4 levels were unchanged [134]. These findings are in contrast to the evaluation of subacute and cumulative effects in lead smelter workers, where no thyroidal effects were shown [138].

Lithium is widely used in the treatment of bipolar mental disorders and has known influences on thyroid function [139], and lithium is used in the manufacturing of button and rechargeable batteries, ceramics, and glass. Recently, lithium has been found in ground and drinking water in Argentina, where the urine lithium concentration corresponded to a daily lithium intake of 2–30 mg [140]. Exposure to lithium in drinking water and other sources seem to suppress thyroid function as urinary lithium was found to correlate negatively with T4 and positively with TSH [141].

## 5. Discussion

As discussed above, several groups of EDCs may have thyroid disrupting potential, but only perchlorate and PCBs have been studied in more detail in humans. Perchlorate reduced expectedly thyroid iodine uptake, but so far, no significant effects on circulating thyroid hormones have been found after exposure to environmental levels of either perchlorate, thiocyanate, or nitrite. Most of the other chemicals have still only been studied in animal models, sporadically, in high doses in volunteers or after occupational or accidental exposure, and results are conflicting. However, all the mentioned chemicals can theoretically have thyroid disrupting properties and consequently further studies are needed to clarify the mechanisms and the general consequences of constant environmental exposure to lower doses. Although thyroid disrupting properties were not documented for all chemicals, especially vulnerable groups like pregnant women, foetuses and children of all ages may be more sensitive because of pregnancy- and growth-related added stress on the thyroid gland, in particular for people living in iodine insufficient areas. Most human studies are performed in groups like healthy volunteers, occupationally exposed individuals, or persons living in certain areas and do not include all thyroid relevant factors as life style, preexisting thyroid disease, age groups, or exposure to other EDCs. However, exposure during the foetal and neonatal period is of much concern, as it is a very vulnerable point in central nervous system development, especially in preterm children. Only few studies of the chemicals in question have addressed the issue of health effects on the offspring of exposed subjects. Yet, many of the potential thyroid disrupting chemicals accumulate both in nature and in exposed individuals and may have a negative influence on maternal thyroid function during pregnancy with consequent risk of impaired neurodevelopment of the foetus. While significant exposure to all these chemicals are suspected to affect human thyroid homeostasis, the effects of environmental exposure still remain to be confirmed in humans and, in particular, in vulnerable groups.

Epidemiological studies have reported that pre- and perinatal exposure to PCBs is associated with poorer neurodevelopment in neonates, toddlers and school-age children [142–147]. The influence of PCBs on thyroid function has been suggested as a reasonable explanation for the results although this was not evaluated in detail. PCB correlated negatively to fT4 in pregnant women [148], and therefore, even exposure at background levels could possible disturb foetal development. 

The subjects in human epidemiological studies have always been exposed to many different compounds through different time periods, and it is, therefore, difficult to isolate specific effects of chemicals and their metabolites on functions of the human organism, which is an obvious caveat in concluding from such studies [59]. 

Some studies have been performed in people more intensively exposed due to either occupation, residency in/near contaminated areas [74, 90, 149, 150], accidents [151], or fish consumption [78, 79, 152, 153], but other studies have focused on general population exposures [58, 83, 96]. There may, thus, be several reasons for the divergence in findings. One explanation could be current low exposure after reduction of allowed limits and, therefore, current unmeasurable levels of a chemical that once was present and exerted an effect. Conflicting results may also reflect that findings depend on the choice of biomarkers, detection methods of the examined EDCs, and sample material, for example, in maternal blood, breast milk, cord blood, or child blood. Furthermore the sex of the foetus, comorbidities, and medication as well as a possible influence from combined effects of other EDCs may influence study outcomes [72]. Even in adult populations, there are probably both age and gender differences in responses in an adult population [83]. 

Given that most of the mentioned chemicals have subtle influences on the thyroid axis, in many cases within the normal reference interval, the question is whether or not such subtle changes in, for example, maternal thyroid function can eventually compromise foetal neurological development. The relationship between T4 and TSH is very unique to each human [154], and the variations within each person are much smaller than the variation within a population [155, 156], which is also the case during pregnancy [157, 158]. Comparison with more or less well-defined population-based reference ranges is probably quite irrelevant considering the discrepancy between these large ranges compared to the much narrower intraindividual variations in thyroid hormone levels [155, 156]. In addition, no first-trimester-specific reference ranges for fT4 analog assays currently exist, available commercial analog fT4 assays are unreliable in pregnant women, and fT4 levels are often over- or under-estimated. In these cases, TT4 and free thyroid hormones indexes are more reliable [159]. Consequently, minor, yet real, changes in thyroid hormone levels due to EDC exposure in small human studies may easily be camouflaged by the broad interindividual variation. As human exposure is lifelong, starting during pregnancy and cumulative for persistent chemicals, it is not possible to design human studies evaluating thyroid function within an individual before and after exposure. Even small intervention studies, like the study with triclosan [109], are performed on a preexisting background of chemical exposure to many other compounds simultaneously. 

Despite this individuality of the thyroid function variables, the levels of TSH and thyroid hormones vary greatly during the early stages of life. TSH increases dramatically immediately after birth peaking at 30 minutes, followed by an increase in T4 and T3, where after all hormone levels decrease. Thyroid hormones measured in newborns may be affected by intrapartum stress [67] and even by use of iodine containing antiseptics [160]. Thus, estimation of any influence of thyroid disrupting chemicals on TSH and thyroid hormones during pregnancy, neonatal period, or early childhood should, therefore, allow for exact age as a critical confounder. 

A possible influence of thyroid hormone-induced metabolism and elimination processes of EDCs, such as detoxification in the liver and kidneys, has not been extensively investigated, and further studies should be performed. Other confounding factors in interpretation of the many results include population-specific level of selenium and iodine, since deficiency of these two substances may render the thyroid system more prone to be affected by EDCs. In addition, exposure to EDCs may cause only transient changes in thyroid hormone levels, which cannot be traced afterwards but, nevertheless, may leave permanent effects on the central nervous system if occurring during a developmentally critical phase. Furthermore, measurement of peripheral thyroid hormone concentrations may not reflect a chemical effect on the full thyroid homeostasis (Figure 1). As outlined in this paper, various chemicals may have different and antagonistic or synergistic effects on the thyroid axis. Such effects have also been found in studies of chemicals disrupting reproduction [18, 19].

Finally, it is not possible in association studies to distinguish whether EDCs could act by direct toxic effects or by indirect mechanisms via disrupting the thyroid function. More mechanistic studies are, therefore, warranted in the future.

## 6. Conclusions

The influence of environmental thyroid disrupting chemicals on maternal thyroid function and consequently on foetal development in humans is still difficult to estimate for several reasons. However, for some of the chemicals, in particular perchlorate and PCBs, evidence is emerging that thyroid function is indeed affected by their exposure, and they therefore potentially possess a damaging effect on foetal development. However, many individual factors including the narrow individual set point for thyroid function, interactions with other environmental factors such as exposure to several EDCs, and deficiency of iodine and/or selenium may interfere with study results and thereby complicate conclusions. Furthermore, it is still not clear which specific cognitive functions in childhood, and consequently which methods of testing, would be the most representative when evaluating permanent effects of thyroid dysfunction during development. Further research in this particular field is necessary to ensure optimal health, growth and development of the foetus, but also for subsequent general thyroid health in children and adults. So, while most available evidence indicates detrimental effects of many EDCs on human thyroid function, thereby potentially affecting pregnant women and consequently foetal development, astonishingly few studies can substantiate this suspicion. Since this may appear to be extremely important for foetal neurodevelopment, researchers in the field should be strongly encouraged to continue the efforts to elucidate the mechanisms in order to be able to prevent damage. This may be so much more important since both populations in iodine deficient areas but also in iodine sufficient areas, with high prevalence of autoimmune hypothyroidism in women of the childbearing age, have an increased susceptibility to the thyroid disrupting properties of EDCs. The complexity of the field and the scarcity of current publications should spur researchers to perform large-scale studies including all relevant confounders, thus hopefully allowing for evidence-based regulations and recommendations.

## Figures and Tables

**Table 1:** Thyroid-disrupting properties of perchlorate in human studies on pregnant women, neonates, infants, adolescents, and adults and the effect of perchlorate on iodine contents in breast milk.

Year	Author	*N*	Subjects	Effect	Reference
2005	Tellez et al.	185	Early pregnant women	No effect	[38]
		135	Late pregnant women	No effect	
		162	Newborns	No effect	
2010	Pearce et al.	1641	Pregnant women	No effect	[36]
2000	Brechner et al.	1542	Newborns	↑TSH	[41]
2000	Li et al.	23000	Newborns	No effect	[42]
2007	Amitai et al.	1156	Newborns	No effect	[37]
2000	Crump et al.	9784	Newborns	↓TSH otherwise no effect	[43]
		162	Schoolchildren	No effect	
2006	Blount et al.	350	Iodine deficient women	↓ TT4 ↑TSH	[34]
		697	Iodine sufficient women	↑TSH	
			Men	No effect	
2000	Lawrence et al.	9	Healthy volunteers	No effect	[33]
				↓ thyroid radioiodine up-take	
2002	Greer et al.	8	Healthy volunteers	↓ thyroid radioiodine up-take	[32]
2006	Braverman et al.	13	Healthy volunteers	No effect	[44]
1998	Gibbs et al.	119	Occupationally exposed	No effect	[45]
1999	Lamm et al.	58	Occupationally exposed	No effect	[46]
2005	Braverman et al.	29	Occupationally exposed	↓ thyroid radioiodine up-take	[31]
					
2005	Kirk et al.	36	Lactating women	↓ Iodine in breast milk	[39]
2007	Pearce et al.	57	Lactating women	No effect on iodine in breast milk	[40]

*N*: number, TSH: thyrotropin, TT3: total tri-iodothyronine, TT4: total thyroxine, fT3: free Tri-iodothyronine, fT4: free thyroxine, and TBG: thyroid hormone-binding globulin.

**Table 2:** Thyroid-disrupting properties of polychlorinated biphenyls in human studies on pregnant women, neonates, infants, adolescents, and adults.

Year	Author	*N*	Subjects	Effect	Reference
1994	Koopman-Esseboom et al.	105	Pregnant women	↓ TT3 ↓ TT4	[62]
		105	Infants	↑TSH at 2 weeks and 3 months	
2005	Takser et al.	101	Pregnant women	↓ TT3 ↑TSH	[58]
		92	Cord blood	No effect	
2008	Wilhelm et al.	165	Pregnant women	No effect	[65]
		127	Cord blood	No effect	
2009	Alvarez-Pedrerol et al.	1090	Pregnant women	↓TT3↑fT4	[70]
2009	Dallaire et al.	120	Pregnant women	↑T3	[71]
		95	Cord blood	↓TBG↓ fT4	
		130	Infants, 7 months old	No effect	
2000	Longnecker et al.	160	Cord blood	No effect	[63]
2005	Wang et al.	118	Cord blood	↓T3↓T4	[72]
2008	Dallaire et al.	670	Cord blood	↓TBG	[68]
2008	Herbstman et al.	289	Cord blood,	↓TT4↓fT4	[67]
		265	Neonatal blood spot**	↓TT4	
2007	Chevrier et al.	285	Newborns	↑TSH	[66]
2001	Matsuura et al.	337	Breastfed infants*	No effect	[64]
2003	Ribas-Fito et al.	98	Infants	Trend toward ↑TSH	[61]
2010	Darnerud et al.	150	Infants	↓ TT3	[73]
1999	Osius et al.	320	Children	↓ fT3 ↑TSH	[74]
2000	Steuerwald et al.	182	Children	No effect	[60]
2008	Alvarez-Pedrerol.	259	Children	↓TT3↓fT4	[75]
2005	Hsu et al.	60	Boys	No effect	[69]
2008	Schell et al.	232	Adolescents	↓ fT4↑TSH	[76]
2001	Sala et al.	192	Adults	Trend toward ↑TSH	[77]
2001	Hagmar et al.	110	Adult men	No effect	[78]
2001	Hagmar et al.	182	Adult women	↓ TT3	[79]
2001	Persky et al.	229	Adults	Female: ↓T4,FTI. Men ↓T3-uptake	[80]
2003	Bloom et al.	66	Adults	No effect	[81]
2003	Langer et al.	101	Adults	↑thyroid volume	[82]
2004	Schell et al.	115	Adults	↓ fT4↓ T4 ↑TSH	
2007	Tyruk et al.	2445	Adults	↓TT4, in older persons↑TSH	[83]
2008	Abdelouahab et al.	211	Adults	Female ↓T3; men ↓T4 ↑TSH	[84]
2009	Dallaire et al.	623	Adults	↓ TT3, ↓TBG	[59]

PCBs were measured in blood unless otherwise stated. *PCBs measured in breast milk. **neonatal blood spot at day 18 postpartum.* N*: number, TSH: thyrotropin, TT3: total tri-iodothyronine, TT4: total thyroxine, fT3 free Tri-iodothyronine, fT4: free thyroxine, FTI: free T4 index, and TBG: thyroid hormone-binding globulin.

## Abbreviations

BPA: Bisphenol A
DDT: Dichlorodiphenyltrichlorethane
EDC: Endocrine disrupting chemical
FT3: Free T3
FT4: Free T4
HCB: Hexachlorobenzene
HCG: Human chorionic gonadotropin
IGF-I: Insulin-like growth factor-I
IQ: Intelligence quotient
NIS: Sodium-iodide symporter
NP: Nonylphenol
PCB: Polychlorinated biphenyl
PFC: Perfluorinated chemical
PCP: Pentachlorophenol
RT3: Reverse T3
TCDD: 2,3,7,8-tetrachlorodibenzo-p-dioxin
TBBPA: Tetrabromobisphenol A
TBG: Thyroxine-binding globulin
TPO: Thyroid peroxidase
Tg: Thyroglobulin
TR: Thyroid hormone receptor
TRE: Thyroid hormone response element
TRH: Thyrotropin-releasing hormone
TSH: Thyrotropin
TSH-R: Thyrotropin receptor
TT3: Total T3
TT4: Total T4
TTR: Transthyretin
T3: Tri-iodo-thyronine
T4: Thyroxine
UDPGT: Uridinediphosphate-glucuronyl transferase
UV: Ultraviolet.

## References

[1] Henrichs J, Bongers-Schokking JJ, Schenk JJ (2010). Maternal thyroid function during early pregnancy and cognitive functioning in early childhood: the generation R study. *Journal of Clinical Endocrinology and Metabolism*.

[2] Pop VJ, Brouwers EP, Vader HL, Vulsma T, Van Baar AL, De Vijlder JJ (2003). Maternal hypothyroxinaemia during early pregnancy and subsequent child development: a 3-year follow-up study. *Clinical Endocrinology*.

[3] Haddow JE, Palomaki GE, Allan WC (1999). Maternal thyroid deficiency during pregnancy and subsequent neuropsychological development of the child. *New England Journal of Medicine*.

[4] Zoeller RT, Rovet J (2004). Timing of thyroid hormone action in the developing brain: clinical observations and experimental findings. *Journal of Neuroendocrinology*.

[5] Berbel P, Mestre JL, Santamaría A (2009). Delayed neurobehavioral development in children born to pregnant women with mild hypothyroxinemia during the first month of gestation: the importance of early iodine supplementation. *Thyroid*.

[6] Raymond J, LaFranchi SH (2010). Fetal and neonatal thyroid function: review and summary of significant new findings. *Current Opinion in Endocrinology, Diabetes and Obesity*.

[7] Bernal J (2007). Thyroid hormone receptors in brain development and function. *Nature Clinical Practice Endocrinology and Metabolism*.

[8] de Escobar GM, Obregón MJ, Escobar del Rey F (2004). Maternal thyroid hormones early in prenancy and fetal brain development. *Best Practice and Research: Clinical Endocrinology and Metabolism*.

[9] Abalovich M, Gutierrez S, Alcaraz G, Maccallini G, Garcia A, Levalle O (2002). Overt and subclinical hypothyroidism complicating pregnancy. *Thyroid*.

[10] Allan WC, Haddow JE, Palomaki GE (2000). Maternal thyroid deficiency and pregnancy complications: implications for population screening. *Journal of Medical Screening*.

[11] Glinoer D (1998). The systematic screening and management of hypothyroidism and hyperthyroidism during pregnancy. *Trends in Endocrinology and Metabolism*.

[12] Klein RZ, Haddow JE, Faix JD (1991). Prevalence of thyroid deficiency in pregnant women. *Clinical Endocrinology*.

[13] Glinoer D (2003). Management of hypo- and hyperthyroidism during pregnancy. *Growth Hormone and IGF Research*.

[14] Glinoer D (1998). Thyroid hyperfunction during pregnancy. *Thyroid*.

[15] Pearce EN, Braverman LE (2009). Environmental pollutants and the thyroid. *Best Practice and Research: Clinical Endocrinology and Metabolism*.

[16] Boas M, Main KM, Feldt-Rasmussen U (2009). Environmental chemicals and thyroid function: an update. *Current Opinion in Endocrinology, Diabetes and Obesity*.

[17] Boas M, Feldt-Rasmussen U, Skakkebæk NE, Main KM (2006). Environmental chemicals and thyroid function. *European Journal of Endocrinology*.

[18] Hass U, Scholze M, Christiansen S (2007). Combined exposure to anti-androgens exacerbates disruption of sexual differentiation in the rat. *Environmental health perspectives*.

[19] Christiansen S, Scholze M, Dalgaard M (2009). Synergistic disruption of external male sex organ development by a mixture of four antiandrogens. *Environmental Health Perspectives*.

[20] Chan SY, Vasilopoulou E, Kilby MD (2009). The role of the placenta in thyroid hormone delivery to the fetus. *Nature Clinical Practice Endocrinology and Metabolism*.

[21] Koopdonk-kool JM, De Vijlder JJM, Veenboer GJM (1996). Type II and type III deiodinase activity in human placenta as a function of gestational age. *Journal of Clinical Endocrinology and Metabolism*.

[22] Kilby MD, Barber K, Hobbs E, Franklyn JA (2005). Thyroid hormone action in the placenta. *Placenta*.

[23] Horacek J, Spitalnikova S, Dlabalova B (2010). Universal screening detects two-times more thyroid disorders in early pregnancy than targeted high-risk case finding. *European Journal of Endocrinology*.

[24] Wang W, Teng W, Shan Z (2011). The prevalence of thyroid disorders during early pregnancy in China: tshe benefits of universal screening in the first trimester of pregnancy. *European Journal of Endocrinology*.

[25] Abalovich M, Amino N, Barbour LA (2007). Management of thyroid dysfunction during pregnancy and postpartum: an Endocrine Society Clinical Practice Guideline. *Journal of Clinical Endocrinology and Metabolism*.

[26] Kooistra L, Crawford S, Van Baar AL, Brouwers EP, Pop VJ (2006). Neonatal effects of maternal hypothyroxinemia during early pregnancy. *Pediatrics*.

[27] Vermiglio F, Lo Presti VP, Moleti M (2004). Attention deficit and hyperactivity disorders in the offspring of mothers exposed to mild-moderate iodine deficiency: a possible novel iodine deficiency disorder in developed countries. *Journal of Clinical Endocrinology and Metabolism*.

[28] Dasgupta PK, Martinelango PK, Jackson WA (2005). The origin of naturally occurring perchlorate: the role of atmospheric processes. *Environmental Science and Technology*.

[29] Martino E, Aghini-Lombardi F, Mariotti S (1986). Treatment of amiodarone associated thyrotoxicosis by simultaneous administration of potassium perchlorate and methimazole. *Journal of Endocrinological Investigation*.

[30] Tonacchera M, Pinchera A, Dimida A (2004). Relative potencies and additivity of perchlorate, thiocyanate, nitrate, and iodide on the inhibition of radioactive iodide uptake by the human sodium iodide symporter. *Thyroid*.

[31] Braverman LE, He X, Pino S (2005). The effect of perchlorate, thiocyanate, and nitrate on thyroid function in workers exposed to perchlorate long-term. *Journal of Clinical Endocrinology and Metabolism*.

[32] Greer MA, Goodman G, Pleus RC, Greer SE (2002). Health effects perchlorate contamination: the dose response for inhibition of thyroidal radioiodine uptake in humans. *Environmental Health Perspectives*.

[33] Lawrence JE, Lamm SH, Pino S, Richman K, Braverman LE (2000). The effect of short-term low-dose perchlorate on various aspects of thyroid function. *Thyroid*.

[34] Blount BC, Pirkle JL, Osterloh JD, Valentin-Blasini L, Caldwell KL (2006). Urinary perchlorate and thyroid hormone levels in adolescent and adult men and women living in the United States. *Environmental Health Perspectives*.

[35] Steinmaus C, Miller MD, Howd R (2007). Impact of smoking and thiocyanate on perchlorate and thyroid hormone associations in the 2001-2002 National Health and Nutrition Examination Survey. *Environmental Health Perspectives*.

[36] Pearce EN, Lazarus JH, Smyth PPA (2010). Perchlorate and thiocyanate exposure and thyroid function in first-trimester pregnant women. *Journal of Clinical Endocrinology and Metabolism*.

[37] Amitai Y, Winston G, Sack J (2007). Gestational exposure to high perchlorate concentrations in drinking water and neonatal thyroxine levels. *Thyroid*.

[38] Téllez RT, Chacón PM, Abarca CR (2005). Long-term environmental exposure to perchlorate through drinking water and thyroid function during pregnancy and the neonatal period. *Thyroid*.

[39] Kirk AB, Martinelango PK, Tian K, Dutta A, Smith EE, Dasgupta PK (2005). Perchlorate and iodide in dairy and breast milk. *Environmental Science and Technology*.

[40] Pearce EN, Leung AM, Blount BC (2007). Breast milk iodine and perchlorate concentrations in lactating Boston-area women. *Journal of Clinical Endocrinology and Metabolism*.

[41] Brechner RJ, Parkhurst GD, Humble WO, Brown MB, Herman WH (2000). Ammonium perchlorate contamination of colorado river drinking water is associated with abnormal thyroid function in newborns in Arizona. *Journal of Occupational and Environmental Medicine*.

[42] Li Z, Li FX, Byrd D (2000). Neonatal thyroxine level and perchlorate in drinking water. *Journal of Occupational and Environmental Medicine*.

[43] Crump C, Michaud P, Téllez R (2000). Does perchlorate in drinking water affect thyroid function in newborns or school-age children?. *Journal of Occupational and Environmental Medicine*.

[44] Braverman LE, Pearce EN, He X (2006). Effects of six months of daily low-dose perchlorate exposure on thyroid function in healthy volunteers. *Journal of Clinical Endocrinology and Metabolism*.

[45] Gibbs JP, Ahmad R, Crump KS (1998). Evaluation of a population with occupational exposure to airborne ammonium perchlorate for possible acute or chronic effects on thyroid function. *Journal of Occupational and Environmental Medicine*.

[46] Lamm SH, Braverman LE, Li FX, Richman K, Pino S, Howearth G (1999). Thyroid health status of ammonium perchlorate workers: a cross-sectional occupational health study. *Journal of Occupational and Environmental Medicine*.

[47] Dohán O, Portulano C, Basquin C, Reyna-Neyra A, Amzel LM, Carrasco N (2007). The Na+/I- symporter (NIS) mediates electroneutral active transport of the environmental pollutant perchlorate. *Proceedings of the National Academy of Sciences of the United States of America*.

[48] Dorea JG (2004). Maternal thiocyanate and thyroid status during breast-feeding. *Journal of the American College of Nutrition*.

[49] Vanderpas J (2006). Nutritional epidemiology and thyroid hormone metabolism. *Annual review of nutrition*.

[50] Tajtáková M, Semanová Z, Tomková Z (2006). Increased thyroid volume and frequency of thyroid disorders signs in schoolchildren from nitrate polluted area. *Chemosphere*.

[51] Gatseva PD, Argirova MD (2008). High-nitrate levels in drinking water may be a risk factor for thyroid dysfunction in children and pregnant women living in rural Bulgarian areas. *International Journal of Hygiene and Environmental Health*.

[52] Gatseva PD, Argirova MD (2008). Iodine status and goitre prevalence in nitrate-exposed schoolchildren living in rural Bulgaria. *Public Health*.

[53] Below H, Zöllner H, Völzke H, Kramer A (2008). Evaluation of nitrate influence on thyroid volume of adults in a previously iodine-deficient area. *International Journal of Hygiene and Environmental Health*.

[54] Ulbrich B, Stahlmann R (2004). Developmental toxicity of polychlorinated biphenyls (PCBs): a systematic review of experimental data. *Archives of Toxicology*.

[55] Purkey HE, Palaninathan SK, Kent KC (2004). Hydroxylated polychlorinated biphenyls selectively bind transthyretin in blood and inhibit amyloidogenesis: rationalizing rodent PCB toxicity. *Chemistry and Biology*.

[56] Miyazaki W, Iwasaki T, Takeshita A, Kuroda Y, Koibuchi N (2004). Polychlorinated biphenyls suppress thyroid hormone receptor-mediated transcription through a novel mechanism. *Journal of Biological Chemistry*.

[57] Kitamura S, Jinno N, Suzuki T (2005). Thyroid hormone-like and estrogenic activity of hydroxylated PCBs in cell culture. *Toxicology*.

[58] Takser L, Mergler D, Baldwin M, de Grosbois S, Smargiassi A, Lafond J (2005). Thyroid hormones in pregnancy in relation to environmental exposure to organochlorine compounds and mercury. *Environmental Health Perspectives*.

[59] Dallaire R, Dewailly É, Pereg D, Dery S, Ayotte P (2009). Thyroid function and plasma concentrations of polyhalogenated compounds in inuit adults. *Environmental Health Perspectives*.

[60] Steuerwald U, Weihe P, Jørgensen PJ (2000). Maternal seafood diet, methylmercury exposure, and neonatal neurologic function. *Journal of Pediatrics*.

[61] Ribas-Fitó N, Sala M, Cardo E (2003). Organochlorine compounds and concentrations of thyroid stimulating hormone in newborns. *Occupational and Environmental Medicine*.

[62] Koopman-Esseboom C, Morse DC, Weisglas-Kuperus N (1994). Effects of dioxins and polychlorinated biphenyls on thyroid hormone status of pregnant women and their infants. *Pediatric Research*.

[63] Longnecker MP, Gladen BC, Patterson DG, Rogan WJ (2000). Polychlorinated biphenyl (PCB) exposure in relation to thyroid hormone levels in neonates. *Epidemiology*.

[64] Matsuura N, Uchiyama T, Tada H (2001). Effects of dioxins and polychlorinated biphenyls (PCBs) on thyroid function in infants born in Japan—the second report from research on environmental health. *Chemosphere*.

[65] Wilhelm M, Wittsiepe J, Lemm F (2008). The Duisburg birth cohort study: influence of the prenatal exposure to PCDD/Fs and dioxin-like PCBs on thyroid hormone status in newborns and neurodevelopment of infants until the age of 24 months. *Mutation Research*.

[66] Chevrier J, Eskenazi B, Bradman A, Fenster L, Barr DB (2007). Associations between prenatal exposure to polychlorinated biphenyls and neonatal thyroid-stimulating hormone levels in a Mexican-American population, Salinas Valley, California. *Environmental Health Perspectives*.

[67] Herbstman JB, Sjödin A, Apelberg BJ (2008). Birth delivery mode modifies the associations between prenatal polychlorinated biphenyl (PCB) and polybrominated diphenyl ether (PBDE) and neonatal thyroid hormone levels. *Environmental Health Perspectives*.

[68] Dallaire R, Dewailly É, Ayotte P, Muckle G, Laliberté C, Bruneau S (2008). Effects of prenatal exposure to organochlorines on thyroid hormone status in newborns from two remote coastal regions in Québec, Canada. *Environmental Research*.

[69] Hsu PC, Lai TJ, Guo NW, Lambert GH, Guo YL (2005). Serum hormones in boys prenatally exposed to polychlorinated biphenyls and dibenzofurans. *Journal of Toxicology and Environmental Health, Part A*.

[70] Alvarez-Pedrerol M, Guxens M, Ibarluzea J (2009). Organochlorine compounds, iodine intake, and thyroid hormone levels during pregnancy. *Environmental Science and Technology*.

[71] Dallaire R, Muckle G, Dewailly É (2009). Thyroid hormone levels of pregnant inuit women and their infants exposed to environmental contaminants. *Environmental Health Perspectives*.

[72] Wang SL, Su PH, Jong SB, Guo YL, Chou WL, Päpke O (2005). In utero exposure to dioxins and polychlorinated biphenyls and its relations to thyroid function and growth hormone in newborns. *Environmental Health Perspectives*.

[73] Darnerud PO, Lignell S, Glynn A, Aune M, Törnkvist A, Stridsberg M (2010). POP levels in breast milk and maternal serum and thyroid hormone levels in mother-child pairs from Uppsala, Sweden. *Environment International*.

[74] Osius N, Karmaus W, Kruse H, Witten J (1999). Exposure to polychlorinated biphenyls and levels of thyroid hormones in children. *Environmental Health Perspectives*.

[75] Álvarez-Pedrerol M, Ribas-Fitó N, Torrent M, Carrizo D, Grimalt JO, Sunyer J (2008). Effects of PCBs, p,p′-DDT, p,p′-DDE, HCB and *β*-HCH on thyroid function in preschool children. *Occupational and Environmental Medicine*.

[76] Schell LM, Gallo MV, Denham M, Ravenscroft J, DeCaprio AP, Carpenter DO (2008). Relationship of thyroid hormone levels to levels of polychlorinated biphenyls, lead, p,p’-DDE, and other toxicants in Akwesasne Mohawk Youth. *Environmental Health Perspectives*.

[77] Sala M, Sunyer J, Herrero C, To-Figueras J, Grimalt J (2001). Association between serum concentrations of hexachlorobenzene and polychlorobiphenyls with thyroid hormone and liver enzymes in a sample of the general population. *Occupational and Environmental Medicine*.

[78] Hagmar L, Björk J, Sjödin A, Bergman A, Erfurth EM (2001). Plasma levels of persistent organohalogens and hormone levels in adult male humans. *Archives of Environmental Health*.

[79] Hagmar L, Rylander L, Dyremark E, Klasson-Wehler E, Erfurth EM (2001). Plasma concentrations of persistent organochlorines in relation to thyrotropin and thyroid hormone levels in women. *International Archives of Occupational and Environmental Health*.

[80] Persky V, Turyk M, Anderson HA (2001). The effects of PCB exposure and fish consumption on endogenous hormones. *Environmental Health Perspectives*.

[81] Bloom MS, Weiner JM, Vena JE, Beehler GP (2003). Exploring associations between serum levels of select organochlorines and thyroxine in a sample of New York state sportsmen: the New York State Angler Cohort Study. *Environmental Research*.

[82] Langer P, Kočan A, Tajtáková M (2003). Possible effects of polychlorinated biphenyls and organochlorinated pesticides on the thyroid after long-term exposure to heavy environmental pollution. *Journal of Occupational and Environmental Medicine*.

[83] Turyk ME, Anderson HA, Persky VW (2007). Relationships of thyroid hormones with polychlorinated biphenyls, dioxins, furans, and DDE in adults. *Environmental Health Perspectives*.

[84] Abdelouahab N, Mergler D, Takser L (2008). Gender differences in the effects of organochlorines, mercury, and lead on thyroid hormone levels in lakeside communities of Quebec (Canada). *Environmental Research*.

[85] Van Der Plas SA, Lutkeschipholt I, Spenkelink B, Brouwer A (2001). Effects of subchronic exposure to complex mixtures of dioxin-like and non-dioxin-like polyhalogenated aromatic compounds on thyroid hormone and vitamin A levels in female Sprague-Dawley rats. *Toxicological Sciences*.

[86] Viluksela M, Raasmaja A, Lebofsky M, Stahl BU, Rozman KK (2004). Tissue-specific effects of 2,3,7,8-tetrachlorodibenzo-p-dioxin (TCDD) on the activity of 5′-deiodinases I and II in rats. *Toxicology Letters*.

[87] Nishimura N, Miyabara Y, Sato M, Yonemoto J, Tohyama C (2002). Immunohistochemical localization of thyroid stimulating hormone induced by a low oral dose of 2,3,7,8-tetrachlorodibenzo-p-dioxin in female Sprague-Dawley rats. *Toxicology*.

[88] Crofton KM, Craft ES, Hedge JM (2005). Thyroid-hormone-disrupting chemicals: evidence for dose-dependent additivity or synergism. *Environmental Health Perspectives*.

[89] Kakeyama M, Tohyama C (2003). Developmental neurotoxicity of dioxin and its related compounds. *Industrial Health*.

[90] Pavuk M, Schecter AJ, Akhtar FZ, Michalek JE (2003). Serum 2,3,7,8-tetrachlorodibenzo-p-dioxin (TCDD) levels and thyroid function in Air Force veterans of the Vietnam War. *Annals of Epidemiology*.

[91] Su PH, Chen JY, Chen JW, Wang SL (2010). Growth and thyroid function in children with in utero exposure to dioxin: a 5-year follow-up study. *Pediatric Research*.

[92] Goodman JE, Kerper LE, Boyce CP, Prueitt RL, Rhomberg LR (2010). Weight-of-evidence analysis of human exposures to dioxins and dioxin-like compounds and associations with thyroid hormone levels during early development. *Regulatory Toxicology and Pharmacology*.

[93] Latini G (2005). Monitoring phthalate exposure in humans. *Clinica Chimica Acta*.

[94] Green R, Hauser R, Calafat AM (2005). Use of di(2-ethylhexyl) phthalate-containing medical products and urinary levels of mono(2-ethylhexyl) phthalate in neonatal intensive care unit infants. *Environmental Health Perspectives*.

[95] Rais-Bahrami K, Nunez S, Revenis ME, Luban NLC, Short BL (2004). Follow-up study of adolescents exposed to Di(2-ethylhexyl) phthalate (DEHP) as neonates on extracorporeal membrane oxygenation (ECMO) support. *Environmental Health Perspectives*.

[96] Meeker JD, Calafat AM, Hauser R (2007). Di(2-ethylhexyl) Phthalate metabolites may alter thyroid hormone levels in men. *Environmental Health Perspectives*.

[97] Huang PC, Kuo PL, Guo YL, Liao PC, Lee CC (2007). Associations between urinary phthalate monoesters and thyroid hormones in pregnant women. *Human Reproduction*.

[98] Boas M, Frederiksen H, Feldt-Rasmussen U (2010). Childhood exposure to phthalates: associations with thyroid function, insulin-like growth factor I, and growth. *Environmental Health Perspectives*.

[99] Meerts IATM, Van Zanden JJ, Luijks EAC (2000). Potent competitive interactions of some brominated flame retardants and related compounds with human transthyretin in Vitro. *Toxicological Sciences*.

[100] Yamauchi K, Ishihara A, Fukazawa H, Terao Y (2003). Competitive interactions of chlorinated phenol compounds with 3,3′,5-triiodothyronine binding to transthyretin: detection of possible thyroid-disrupting chemicals in environmental waste water. *Toxicology and Applied Pharmacology*.

[101] Moriyama K, Tagami T, Akamizu T (2002). Thyroid hormone action is disrupted by bisphenol A as an antagonist. *Journal of Clinical Endocrinology and Metabolism*.

[102] Sun H, Shen OX, Wang XR, Zhou L, Zhen SQ, Chen XD (2009). Anti-thyroid hormone activity of bisphenol A, tetrabromobisphenol A and tetrachlorobisphenol A in an improved reporter gene assay. *Toxicology in Vitro*.

[103] Calafat AM, Ye X, Wong LY, Reidy JA, Needham LL (2008). Exposure of the U.S. population to bisphenol A and 4-tertiary-octylphenol: 2003-2004. *Environmental health perspectives*.

[104] Ye X, Pierik FH, Hauser R (2008). Urinary metabolite concentrations of organophosphorous pesticides, bisphenol A, and phthalates among pregnant women in Rotterdam, the Netherlands: the Generation R study. *Environmental Research*.

[105] Cao J, Guo L-H, Wan B, Wei Y (2011). In vitro fluorescence displacement investigation of thyroxine transport disruption by bisphenol A. *Journal of Environmental Sciences*.

[106] Zoeller RT, Bansal R, Parris C (2005). Bisphenol-A, an environmental contaminant that acts as a thyroid hormone receptor antagonist in vitro, increases serum thyroxine, and alters RC3/neurogranin expression in the developing rat brain. *Endocrinology*.

[107] Calafat AM, Ye X, Wong LY, Reidy JA, Needham LL (2008). Urinary concentrations of triclosan in the U.S. population: 2003-2004. *Environmental Health Perspectives*.

[108] Adolfsson-Erici M, Pettersson M, Parkkonen J, Sturve J (2002). Triclosan, a commonly used bactericide found in human milk and in the aquatic environment in Sweden. *Chemosphere*.

[109] Allmyr M, Panagiotidis G, Sparve E, Diczfalusy U, Sandborgh-Englund G (2009). Human exposure to triclosan via toothpaste does not change cyp3a4 activity or plasma concentrations of thyroid hormones. *Basic and Clinical Pharmacology and Toxicology*.

[110] Jacobs MN, Nolan GT, Hood SR (2005). Lignans, bacteriocides and organochlorine compounds activate the human pregnane X receptor (PXR). *Toxicology and Applied Pharmacology*.

[111] Paul KB, Hedge JM, DeVito MJ, Crofton KM (2010). Developmental triclosan exposure decreases maternal and neonatal thyroxine in rats. *Environmental Toxicology and Chemistry*.

[112] Rodríguez PEA, Sanchez MS (2010). Maternal exposure to triclosan impairs thyroid homeostasis and female pubertal development in wistar rat offspring. *Journal of Toxicology and Environmental Health, Part A*.

[113] Boker LK, Van Der Schouw YT, De Kleijn MJJ, Jacques PF, Grobbee DE, Peeters PHM (2002). Intake of dietary phytoestrogens by Dutch women. *Journal of Nutrition*.

[114] Divi RL, Chang HC, Doerge DR (1997). Anti-thyroid isoflavones from soybean. Isolation, characterization, and mechanisms of action. *Biochemical Pharmacology*.

[115] Doerge DR, Sheehan DM (2002). Goitrogenic and estrogenic activity of soy isoflavones. *Environmental Health Perspectives*.

[116] Messina M, Redmond G (2006). Effects of soy protein and soybean isoflavones on thyroid function in healthy adults and hypothyroid patients: a review of the relevant literature. *Thyroid*.

[117] Chevrier J, Harley KG, Bradman A, Gharbi M, Sjödin A, Eskenazi B (2010). Polybrominated diphenyl ether (PBDE) flame retardants and thyroid hormone during pregnancy. *Environmental Health Perspectives*.

[118] Turyk ME, Persky VW, Imm P, Knobeloch L, Chatterton R, Anderson HA (2008). Hormone disruption by PBDEs in adult male sport fish consumers. *Environmental Health Perspectives*.

[119] Julander A, Karlsson M, Hagström K (2005). Polybrominated diphenyl ethers—plasma levels and thyroid status of workers at an electronic recycling facility. *International Archives of Occupational and Environmental Health*.

[120] Mazdai A, Dodder NG, Abernathy MP, Hites RA, Bigsby RM (2003). Polybrominated diphenyl ethers in maternal and fetal blood samples. *Environmental Health Perspectives*.

[121] Gocmen A, Peters HA, Cripps DJ, Bryan GT, Morris CR (1989). Hexachlorobenzene episode in Turkey. *Biomedical and Environmental Sciences*.

[122] Sandau CD, Ayotte P, Dewailly É, Duffe J, Norstrom RJ (2002). Pentachlorophenol and hydroxylated polychlorinated biphenyl metabolites in umbilical cord plasma of neonates from coastal populations in Québec. *Environmental Health Perspectives*.

[123] Gray LE, Ostby J, Ferrell J (1989). A dose-response analysis of methoxychlor-induced alterations of reproductive development and function in rat. *Fundamental and Applied Toxicology*.

[124] Fort DJ, Guiney PD, Weeks JA (2004). Effect of methoxychlor on various life stages of Xenopus laevis. *Toxicological Sciences*.

[125] Bondy G, Curran I, Doucet J (2004). Toxicity of trans-nonachlor to Sprague-Dawley rats in a 90-day feeding study. *Food and Chemical Toxicology*.

[126] Sinha N, Lal B, Singh TP (1991). Effect of endosulfan on thyroid physiology in the freshwater catfish, Clarias batrachus. *Toxicology*.

[127] Chiba I, Sakakibara A, Goto Y (2001). Negative correlation between plasma thyroid hormone levels and chlorinated hydrocarbon levels accumulated in seals from the coast of Hokkaido, Japan. *Environmental Toxicology and Chemistry*.

[128] Rousset B (1981). Antithyroid effect of a food or drug preservative: 4-hydroxybenzoic acid methyl ester. *Experientia*.

[129] Melzer D, Rice N, Depledge MH, Henley WE, Galloway TS (2010). Association between serum perfluorooctanoic acid (PFOA) and thyroid disease in the U.S. National Health and Nutrition Examination Survey. *Environmental Health Perspectives*.

[130] Olsen GW, Zobel LR (2007). Assessment of lipid, hepatic, and thyroid parameters with serum perfluorooctanoate (PFOA) concentrations in fluorochemical production workers. *International Archives of Occupational and Environmental Health*.

[131] Date K, Ohno K, Azuma Y (2002). Endocrine-disrupting effects of styrene oligomers that migrated from polystyrene containers into food. *Food and Chemical Toxicology*.

[132] Santini F, Mantovani A, Cristaudo A (2008). Thyroid function and exposure to styrene. *Thyroid*.

[133] Dundar B, Öktem F, Arslan MK (2006). The effect of long-term low-dose lead exposure on thyroid function in adolescents. *Environmental Research*.

[134] Singh B, Chandran V, Bandhu HK (2000). Impact of lead exposure on pituitary-thyroid axis in humans. *BioMetals*.

[135] Huseman CA, Moriarty CM, Angle CR (1987). Childhood lead toxicity and impaired release of thyrotropin-stimulating hormone. *Environmental Research*.

[136] Siegel M, Forsyth B, Siegel L, Cullen MR (1989). The effect of lead on thyroid function in children. *Environmental Research*.

[137] Robins JM, Cullen MR, Kayne RD (1983). Depressed thyroid indexes associated with occupational exposure to inorganic lead. *Archives of Internal Medicine*.

[138] Schumacher C, Brodkin CA, Alexander B (1998). Thyroid function in lead smelter workers: absence of subacute or cumulative effects with moderate lead burdens. *International Archives of Occupational and Environmental Health*.

[139] Grandjean EM, Aubry JM (2009). Lithium: Updated human knowledge using an evidence-based approach: part III: clinical safety. *CNS Drugs*.

[140] Concha G, Broberg K, Grandér M, Cardozo A, Palm B, Vahter M (2010). High-level exposure to lithium, boron, cesium, and arsenic via drinking water in the Andes of Northern Argentina. *Environmental Science and Technology*.

[141] Broberg K, Concha G, Engstrom K, Lindvall M, Grander M, Vahter M (2011). Lithium in drinking water and thyroid function. *Environmental Health Perspectives*.

[142] Grandjean P, Weihe P, Burse VW (2001). Neurobehavioral deficits associated with PCB in 7-year-old children prenatally exposed to seafood neurotoxicants. *Neurotoxicology and Teratology*.

[143] Jacobson JL, Jacobson SW (1996). Dose-response in perinatal exposure to polychlorinated biphenyls (PCBs): the Michigan and North Carolina cohort studies. *Toxicology and Industrial Health*.

[144] Koopman-Esseboom C, Weisglas-Kuperus N, De Ridder MAJ, Van Der Paauw CG, Tuinstra LGMT, Sauer PJJ (1996). Effects of polychlorinated biphenyl/dioxin exposure and feeding type on infantś mental and psychomotor development. *Pediatrics*.

[145] Rogan WJ, Gladen BC (1991). PCBs, DDE, and child development at 18 and 24 months. *Annals of Epidemiology*.

[146] Schantz SL, Levin ED, Bowman RE, Heironimus MP, Laughlin NK (1989). Effects on perinatal PCB exposure on discrimination-reversal learning in monkeys. *Neurotoxicology and Teratology*.

[147] Widholm JJ, Villareal S, Seegal RF, Schantz SL (2004). Spatial alternation deficits following developmental exposure to Aroclor 1254 and/or methylmercury in rats. *Toxicological Sciences*.

[148] Chevrier J, Eskenazi B, Holland N, Bradman A, Barr DB (2008). Effects of exposure to polychlorinated biphenyls and organochlorine pesticides on thyroid function during pregnancy. *American Journal of Epidemiology*.

[149] Langer P, Tajtáková M, Fodor G (1998). Increased thyroid volume and prevalence of thyroid disorders in an area heavily polluted by polychlorinated biphenyls. *European Journal of Endocrinology*.

[150] Calvert GM, Sweeney MH, Deddens J, Wall DK (1999). Evaluation of diabetes mellitus, serum glucose, and thyroid function among United States workers exposed to 2,3,7,8-tetrachlorodibenzo-p-dioxin. *Occupational and Environmental Medicine*.

[151] Murai K, Okamura K, Tsuji H (1987). Thyroid function in “Yusho” patients exposed to polychlorinated biphenyls (PCB). *Environmental Research*.

[152] Langer P, Tajtáková M, Kočan A (2007). Thyroid ultrasound volume, structure and function after long-term high exposure of large population to polychlorinated biphenyls, pesticides and dioxin. *Chemosphere*.

[153] Turyk ME, Anderson HA, Freels S (2006). Associations of organochlorines with endogenous hormones in male Great Lakes fish consumers and nonconsumers. *Environmental Research*.

[154] Spencer CA, LoPresti JS, Patel A (1990). Applications of a new chemiluminometric thyrotropin assay to subnormal measurement. *Journal of Clinical Endocrinology and Metabolism*.

[155] Feldt-Rasmussen U, Petersen PH, Blaabjerg O, Horder M (1980). Long-term variability in serum thyroglobulin and thyroid related hormones in healthy subjects. *Acta Endocrinologica*.

[156] Andersen S, Pedersen KM, Bruun NH, Laurberg P (2002). Narrow individual variations in serum T4 and T3 in normal subjects: a clue to the understanding of subclinical thyroid disease. *Journal of Clinical Endocrinology and Metabolism*.

[157] Boas M, Forman JL, Juul A (2009). Narrow intra-individual variation of maternal thyroid function in pregnancy based on a longitudinal study on 132 women. *European Journal of Endocrinology*.

[158] Feldt-Rasmussen U, Bliddal S, Rasmussen ÅK, Boas M, Hilsted L, Main K (2011). Challenges in interpretation of thyroid function tests in pregnant woman with autoimmune thyroid disease. *Journal of Thyroid Research*.

[159] Lee RH, Spencer CA, Mestman JH (2009). Free T4 immunoassays are flawed during pregnancy. *American Journal of Obstetrics and Gynecology*.

[160] Copeland DL, Sullivan KM, Houston R (2002). Comparison of neonatal thyroid-stimulating hormone levels and indicators of iodine deficiency in school children. *Public Health Nutrition*.

